# Detection of Intratumoral Susceptibility Signals Using T2*-Weighted Gradient Echo MRI in Patients with Clear Cell Renal Cell Carcinoma

**DOI:** 10.1371/journal.pone.0079597

**Published:** 2013-11-12

**Authors:** Shengnan Yu, Jianguo Qiu, Jinggang Zhang, Liang Pan, Shijun Xing, Lijun Zhang

**Affiliations:** Department of Radiology, The First People's Hospital of Changzhou, Changzhou, Jiangsu, China; The University of Chicago, United States of America

## Abstract

**Objective:**

To retrospectively evaluate whether T2*-weighted imaging can be used to grade clear cell renal cell carcinomas (ccRCC) based on intratumoral susceptibility signals (ISSs).

**Materials and Methods:**

MR imaging from 37 patients with pathologically-proven ccRCCs was evaluated. ISSs on T2*WI were classified as linear or conglomerated linear structures (type I) and dot-like or patchy foci (type II). Two radiologists assessed the likelihood of the presence of ISS, dominant structure of ISS and ratio of ISS area to tumor area. Results were analyzed by nonparametric Mann-Whitney test.

**Results:**

ISSs were seen in all patients except for four patients with low-grade ccRCCs and two patients with high-grade ccRCCs. There was no significant difference of the likelihood of the presence of ISS between low- and high-grade ccRCCs. More type I ISSs and less type II ISSs were predictive of low-grade tumors, whereas more conspicuity type II ISSs correlated with higher occurrence of high-grade tumors (*P*<0.05). The ratio of ISS area to tumor area was also significantly higher for the high-grade group (1.27±0.79) than that for the low-grade group (0.81±0.40) (*P*<0.05).

**Conclusion:**

ISSs on T2*-weighted gradient-echo MR images can help grade ccRCCs before operations.

## Introduction

Renal cell carcinoma (RCC) is a primary malignancy of the kidney that arises from the renal parenchyma. It is a form of adenocarcinoma, constituting upwards to 90% of primary renal malignancies in human adults [1]. In the United States, the incidence of RCC has continued to rise, with much of the rise being attributed to advanced imaging techniques and earlier detection [1]. Clear cell RCC (ccRCC) constitutes the majority of RCCs. The diagnosis of ccRCC depends on pathological analysis of suspected lesions. Histopathological grade of ccRCC is an independent factor that predicts prognosis and survival [2].

Fuhrman et al. [3] proposed a grading system for RCC based on the morphology of nuclei and nucleoli. This grading system has been widely used to predict the prognosis of patients with RCC and can help assess tumor aggressiveness [4], [5]. Correlations between pathological grades of ccRCC and tumor size have been reported in previous studies [6]–[8]. Unfortunately, the correlation between tumor size and pathological grade is still controversial [9].

T2*- based MR imaging is sensitive to the magnetic field in homogeneities and can be used to explore the magnetic susceptibility difference among various tissues. It is particularly useful in depicting pathological conditions such as cerebral hemorrhage, arteriovenous malformations, cavernomas, as well as hemorrhage in tumors [10]. In recent studies, T2*-weighted MRI sequence was used to identify abdominal tumors [11]–[12]. Intratumoral hemorrhage and microvascularity are the most commonly histopathological conditions which can cause intratumoral susceptibility signals (ISS) on MRI. In previous studies, magnetic susceptibility signals in the lesion on MRI were used to grade gliomas [13]–[15].

To the best of our knowledge, the correlation between pathological grades and ISSs on T2*-weighted imaging (T2*WI) in ccRCC has not been studied before. This study aims to explore the feasibility of T2*WI in differentiating pathological grades of ccRCCs.

## Materials and Methods

### Study Patients

This retrospective study was approved by the Institutional Review Board Committees of The First People's Hospital of Changzhou with waivers of informed consent and was conducted according to the principles expressed in the Declaration of Helsinki.

The inclusion criteria for patients were as follows: Total or partial nephrectomy was performed in our hospital from October 2011 to September 2012. MR scans were undergone preoperatively. Pathological results confirmed the diagnosis of ccRCCs. One patient was excluded because of obvious breathing artifacts on MR imaging. Finally, 37 patients (23 men and 14 women; ranging 21–77 years old; median age, 56 years) were included in our research.

### MR Imaging Technique

All subjects were examined with a standard 12-channel phase array body-matrix coil and 3T systems (MAGNTEOM Verio, Siemens Healthcare, Erlangen, Germany). The MR sequences for all the patients included: (a) coronal breath-hold half acquisition single-shot turbo spin echo (HASTE) T2-weighted imaging (T2WI) (TR/TE, 800/91 ms; field of view, 380 mm×380 mm; matrix size, 117×256; slice thickness, 4 mm; gap 1.95 mm; flip angle, 160°; bandwidth, 781 Hz/pixel); (b) transversal gradient-recalled-echo (GRE) T1-weighted imaging (T1WI) (TR/TE, 161/2.5 ms; field of view, 285 mm×380 mm; matrix size, 180×320; slice thickness, 5 mm; slice gap 1.0 mm; flip angle, 70°; bandwidth, 270 Hz/pixel); (c) transversal HASTE T2WI (TR/TE, 700/96 ms; field of view, 285 mm×380 mm; matrix size, 168×320; slice thickness, 5 mm; gap 1.0 mm; flip angle, 150°; bandwidth, 488 Hz/pixel); and (d) a multi-breath-hold, transversal single-echo GRE T2*WI (TR/TE, 336/9.76 ms; field of view, 270 mm×360 mm; matrix size, 163×256; slice thickness, 5 mm; gap 1.0 mm; flip angle, 30; an acquisition time of 75 seconds including three breath-holds of 55 seconds and two breaks of 10 seconds in between).

### Data Analysis

Two readers, who were blinded to histopathological results, evaluated all the studies in consensus. All images were analyzed on a commercial workstation (Syngo, Siemens Healthcare, Erlangen, Germany).

The likelihood of presence of ISSs was used to evaluate whether ISSs were present on T2*WI and was scored by using a 4-point grading system [14] : 0 = no ISSs, 1 =  a focus of ISSs less than 0.5 cm in largest dimension in transverse planes, 2 =  a focus of ISSs 0.5–1.0 cm in largest dimension, 3 =  a focus of ISSs greater than 1.0 cm in largest dimension.

ISSs were classified into two different type based on the characteristic of their morphology. Type I was defined as fine linear or conglomerated linear hypointensity structures in transverse planes or cylindrical hypointentsity that could be followed on contiguous images. Type II was defined as intratumorl dot-like or patchy (conglomerated dots) hypointensity foci, which were larger than 5 mm in diameter [16]. The dominant structure of ISSs was assigned a numerical value according to a scale ranging from 0 to 3 as follows: 0 =  no ISSs in the tumor on T2*WI; 1 =  prominently type I ISSs ; 2 =  type I and II ISSsalmost equally presented in the tumor on T2*WI; 3 =  prominently type II ISSs.

The ratio of ISS area to tumor area was scored by a 3-point grading system: 0 =  no ISSs, 1 =  the ratio less than half of the tumor on any slice, 2 =  the ratio greater than half of the tumor in at least one slice.

### Histopathological Analysis

Histopathology was obtained from all of our enrolled patients' renal masses through radical nephrectomy. According to the Fuhrman criteria [3], each case was classified as grade I, II, III, IV. On the basis of the Fuhrman nuclear grade, all cases were merged into low- (Grade I+ II) and high-grade group (Grade III + IV) [17].

### Statistical analyses

Statistical analysis was performed using SPSS (version 17.0; SPSS, Chicago, IL, USA). Results were presented as mean ± SD. The differences of the likelihood of presence of ISSs, dominant structure of ISSs and ratio of ISSs area on T2*WI between low- and high-grade tumors were compared by nonparametric Mann-Whitney test. A difference of *P*<0.05 was considered significant.

## Results

### Histopathological Results

In all patients, Fuhrman grade I was found in 12 of 37 (32%); grade II, in 14 of 37 (38%); grade III, in 8 of 37 (22%); and grade IV, in 3 of 37 (8%) patients. Low-grade group contained 26 cases and high-grade group contained 11 cases.

### MRI Results

In low-grade ccRCCs, ISSs were seen in 21 patients on T2*WI. In high-grade ccRCCs, ISSs were seen in 9 patients. Mean scores of the likelihood of presence of ISSs were 1.69±1.09 for low-grade ccRCCs and 2.18±1.17 for high-grade ccRCCs, respectively. No significant differences of the likelihood of presence of ISSs on T2*WI between low- and high-grade ccRCCs were seen (*P*>0.05).

**Figure 1:**
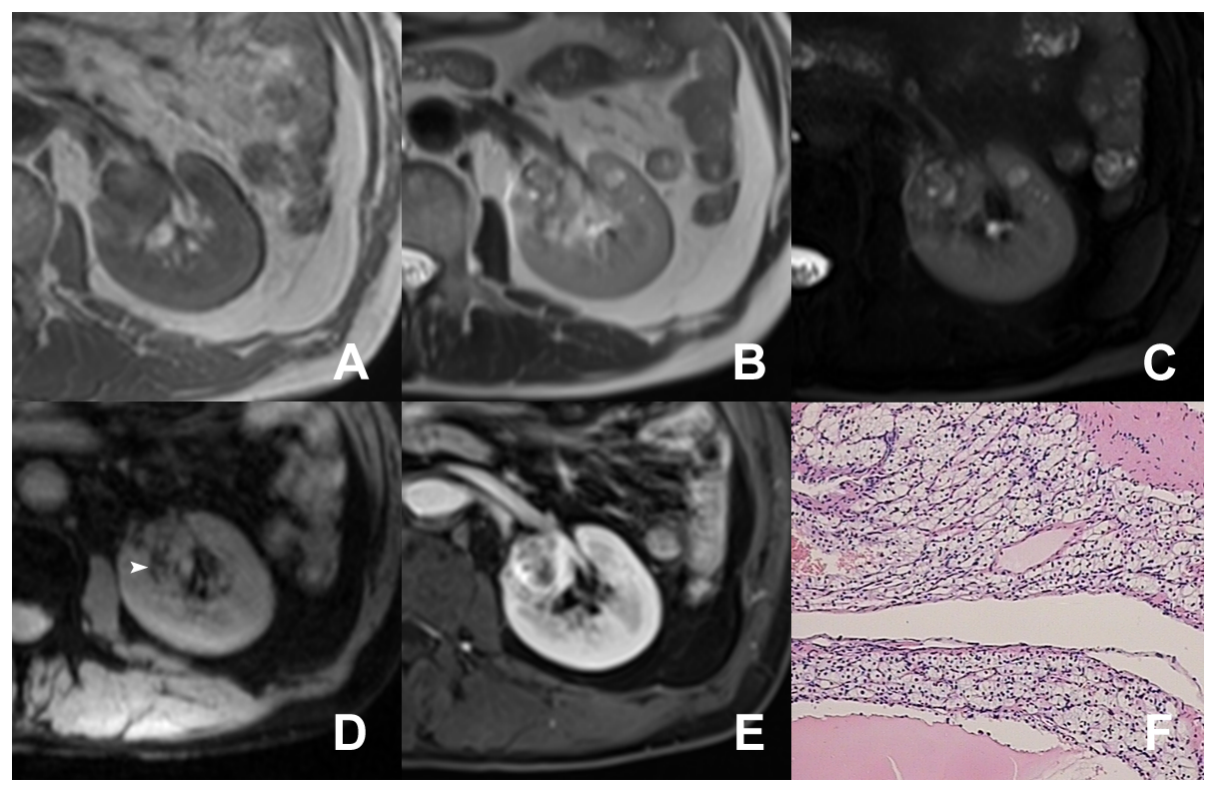
A patient with low-grade ccRCC (Grade I). In the left kidney, a small mass with hypointensity is seen on T1WI (A). On T2WI (B), it shows heterogeneous hyperintensity. Some areas of necrosis showed high signal intense on fat-suppressed T2WI (C). On T2*WI (D), some linear susceptibility signals are seen (arrow head). After administrating contrast agents, the tumor reveal avid enhancement (E). Photomicrograph shows small (<10 µm), hyperchromatic, and round nuclei without visible nucleoli (hematoxylin and eosin ×200) (F).

**Figure 2:**
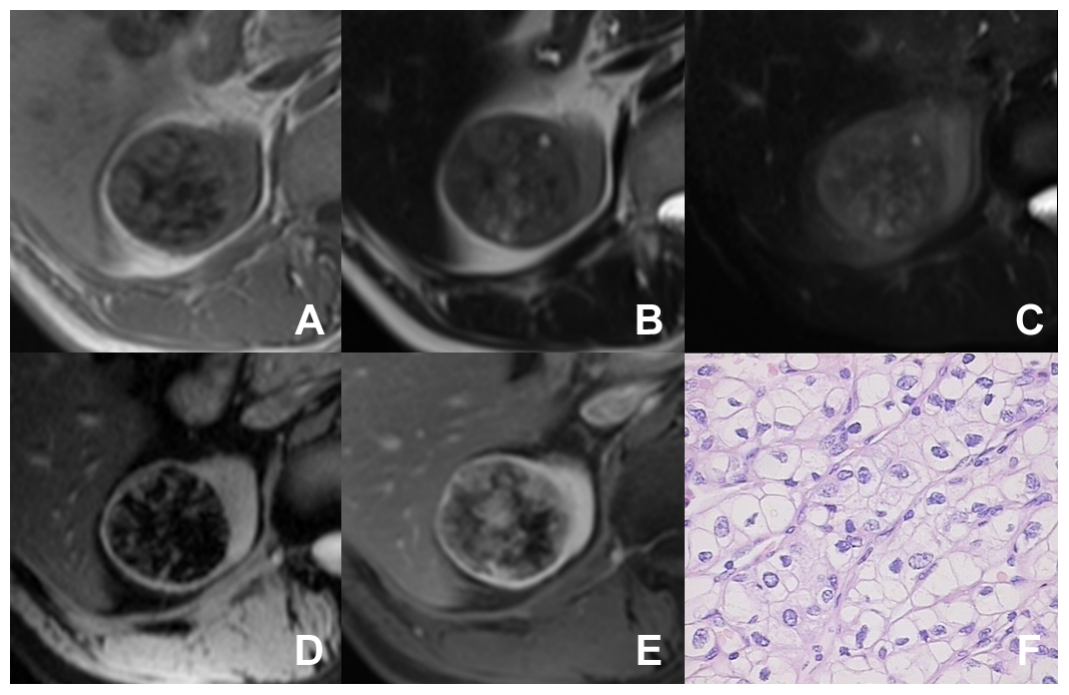
A patient with high-grade ccRCC (Grade III). A mass in the right kidney appears heterogeneous signal intense on T1WI (A) and T2WI (B). On T1WI and T2WI, some patchy hypointensity lesions are seen, which represent hemosiderin deposition. On fat-suppressed T2WI (C), the tumor shows heterogeneous signal intense. Some cystic areas are seen. On T2*WI (D), the hypointensity area appears more extensive than those on T1WI and T2WI. After enhancement, the tumor is enhanced heterogeneously (E). Photomicrograph shows large, irregular nuclei with prominent nucleoli (hematoxylin and eosin ×400) (F).

In low-grade ccRCCs, all patients showed type I ISSs or type I and II ISSs almost equally presented. In high-grade ccRCCs, all patients showed prominently type II ISSs. The morphology of the ISSs on T2*WI in every grade was list in Table 1. Mean scores of dominant structures of ISSs on T2*WI were significantly lower for low-grade ccRCCs (1.19±0.75) (Fig. 1) than those for the high-grade ccRCCs (2.36±1.21) (Fig. 2) (*P*<0.05).

**Table 1:** The morphology of the ISSs on T2*WI.

	Grade I(n = 12)	Grade II(n = 14)	Grade III(n = 8)	Grade IV(n = 3)
No ITSSs	3	2	2	0
Type I ISSs	5	6	0	0
Type II ISSs	0	0	5	3
Type I and II ISSs almost equally presented in the tumor	4	6	1	0

The ratio of ISS area to tumor area on T2*WI was significantly higher for high-grade tumors (1.27±0.79) than that for low-grade tumors (0.81±0.40) (*P*<0.05).

## Discussion

In this study, we evaluated whether ISSs were present in ccRCCs using T2*-weighted GRE MRI. We qualitatively assessed the likelihood of presence of ISSs, as well as morphology of ISSs, with histopathological tumor grading. Additionally, we quantitatively assessed the ratio of ISSs to tumor area, and correlated it with histopathological tumor grading. Our study revealed that there was a significant difference in the dominant morphological structures of ISSs on T2*WI between low-and high-grade ccRCCs, with low-grade tumors having more type I ISSs and less type II ISSs than high grade tumors. We also found a direct correlation of the ratio of ISS to tumor area between low- and high-grade ccRCCs. These results suggest that T2*WI could serve as a useful tool in the process of grading ccRCCs.

Active surveillance (AS) is a new management for patients with renal tumors as well as advanced age and comorbidity [18], [19]. The growth rate of RCC and occurrence of metastases need be taken into consideration to evaluate the feasibility of AS. Fuhrman nuclear grade is the most widely used to determine histological grade of RCC and correlates closely with growth rate and prognosis of ccRCCs. Oda et al. [20] reported a significant relationship between the growth rate of metastatic RCC lesions and the pathological grade of the primary lesion. Kato et al. [21] also suggested a significant difference in growth rate of primary RCCs between lower grade and higher grade tumors. So, if we can predict metastatic likelihood through nuclear grade evaluation with T2*WI, it will help in further evaluation and the planning of treatment in the clinical setting.

The fine needle biopsy is the most common approach to estimate histopathological results of RCC. But the pathological evaluation of RCCs is not always available preoperatively and fine needle biopsy has several disadvantages, including sample errors, insufficient material, and only determining whether the cells are malignant. So a newly effective approach which can predict tumor nuclear grade is very important. T2*WI may provide a solution in these settings, as it will help in the non-invasive evaluation of tumor grade.

ISSs have been proven to be associated with vascular density and blood volume in tumors as well as be used to assess the correlation between the microvascular density and tumor cell proliferation [22]. Some previous studies have reported that intratumoral hemorrhage and microvascularity correlated with pathological grades of gliomas [23], [24]. Bagley et al. [13] was the first to report the correlation between intratumral susceptibility signals and glioma grades. Park et al. classified the intratumoral susceptibility signals into two types based on their morphology [15]. In his study, susceptibility signals only were seen in high-grade gliomas and showed conglomerated mixed fine linear and dot-like structures In this study, our results suggested that the difference of intratumoral susceptibility signals detected on T2*WI was present between low- and high-grade ccRCCs. But our results demonstrated ISSs were present in low-grade ccRCCs. This difference between our results and previous studies might be caused by difference of biological behavior between two types of tumors.

Some earlier studies have reported the correlation between the microvessel count and ccRCC grades [25], [26]. In our study, the score of dominant structure of susceptibility artifacts on T2*WI in low-grade tumors was lower than that in high-grade tumors. Our results demonstrated that more type I ISSs and less type II ISSs were present in low-grade tumors than those in high-grade tumors. This phenomenon can perhaps be explained by a study by Sabo et al. [27], which suggested that low grade RCC tumors have more complex and intact vasculature, as well as less hemorrhage than high grade RCCs.

Our study had the following study limitations. First, the number of patients in our study, especially patients with high-grade ccRCCs, was small. A larger clinical trial with more patients is needed. Second, in our study only ccRCCs were evaluated by using T2*WI because another subtype of RCC was relatively rare and the Fuhrman nuclear grading system has been questioned for some subtypes. Finally, although our study confirmed the feasibility of T2*WI in grading ccRCC, the signal-to-noise (SNR) of images is not very high. Now some third-party software has been introduced to extract the information of magnitude and phase images and generate the susceptibility-weighted images, which have higher SNR and are more sensitive to magnetic susceptibility.

In conclusion, our study indicates that T2*WI can reveal ISSs of ccRCCs. Dominant structures of susceptibility signals and the ratio of susceptibility signals area to tumor area are two important markers for grading ccRCC.
